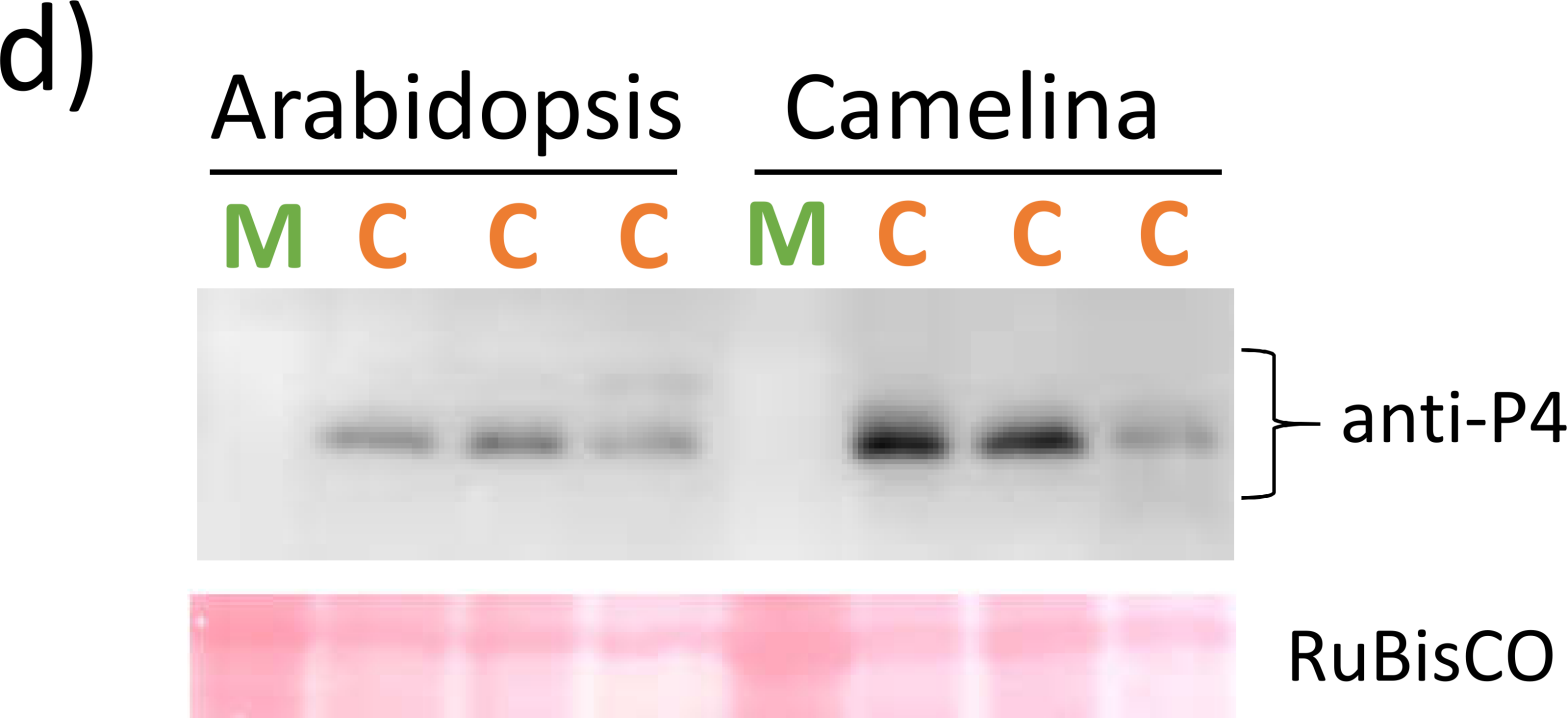# Erratum for Chesnais et al., “Comparative Plant Transcriptome Profiling of *Arabidopsis thaliana* Col-0 and *Camelina sativa* var. *Celine* Infested with *Myzus persicae* Aphids Acquiring Circulative and Noncirculative Viruses Reveals Virus- and Plant-Specific Alterations Relevant to Aphid Feeding Behavior and Transmission”

**DOI:** 10.1128/spectrum.01924-24

**Published:** 2024-09-11

**Authors:** Quentin Chesnais, Victor Golyaev, Amandine Velt, Camille Rustenholz, Véronique Brault, Mikhail M. Pooggin, Martin Drucker

## ERRATUM

Volume 10, no. 4, e00136-22, 2022, https://doi.org/10.1128/spectrum.00136-22. Figure 1d should appear as shown below.

**Fig 1:**