# Supplementary material for: Comparative Plant Transcriptome Profiling of Arabidopsis thaliana Col-0 and Camelina sativa var. Celine Infested with Myzus persicae Aphids Acquiring Circulative and Noncirculative Viruses Reveals Virus- and Plant-Specific Alterations Relevant to Aphid Feeding Behavior and Transmission
Source: Microbiol Spectr. 2022 Jul 20;10(4):e00136-22. doi: 10.1128/spectrum.00136-22 (PMC9430646; doi:10.1128/spectrum.00136-22)
Supplement: Supplemental file 1 — Supplemental material. Download spectrum.00136-22-s0004.pdf, PDF file, 1.0 MB [file spectrum.00136-22-s0004.pdf]

Supplementary files

Provided in this file:

Table S1: Oligonucleotides used for RT-qPCR

Table S2: Aligned reads for transcriptome profiling

Table S3: List of plant genes upregulated by aphid infestation alone (extracted from the RNA-seq data by Annacondia et al. (2021)) but downregulated in plants concomitantly aphid-infested and infected by TuYV or CaMV.

Figure S1. Validation of Illumina RNA-seq expression data by quantitative reverse-transcription PCR (RT-qPCR)

Figure S2: Gene ontology analysis showing the Top 25 GO of deregulated processes

Figure S3: Supplementary heatmaps

Supplementary Sequence Information S1 on CaMV and TuYV

Provided as extra files:

Supplementary_Dataset_S1_Plant_mRNA-seq.xlsx

Supplementary_Dataset_S2_Heatmaps_DEGs_List.xlsx

Supplementary_Dataset_Table_S3.xlsx

Table S1: Oligonucleotides used for RT-qPCR

Gene	Organism	Primers
AT5G25760 (PEX4)	*A. thaliana*	Forward primer<br>TGCAACCTCCTCAAGTTCGA<br>Reverse primer<br>GCAGGACTCCAAGCATTCTT
AT2G18700 (TPS11)	*A. thaliana*	Forward primer<br>AAGTTTTGGGCGATGGGTCA<br>Reverse primer<br>CGAGAACCACTTTCCCACGA
AT1G61800 (GPT2)	*A. thaliana*	Forward primer<br>AGTGTCATTTCTTGATCAGACCATC<br>Reverse primer<br>CCAGTAGCGACACACCTCAAT
AT4G39030 (EDS5)	*A. thaliana*	Forward primer<br>ACCCTAGCGACAAATGACAGC<br>Reverse primer<br>TCACTTGCTCCATTATTAACCTGC
AT1G31280 (AGO2)	*A. thaliana*	Forward primer<br>ATGCTGACAAGGCTGCTTCT<br>Reverse primer<br>CAGAAGACGAAGACGCTCCA
AT4G26530 (FBA5)	*A. thaliana*	Forward primer<br>TTGGTTGCCATTTGGTTGTGT<br>Reverse primer<br>CTGAAGAGGACGAGGATGCC
AT3G26830 (PAD3)	*A. thaliana*	Forward primer<br>AGGGCAAGGAAAATGTCGGT<br>Reverse primer<br>CAGGGGTAAGAGGACGAGGA
AT5G42650 (AOS)	*A. thaliana*	Forward primer<br>TCACGATGGGAGCGATTGAG<br>Reverse primer<br>ACCGTATTGAGCCGTAACCG
AT4G13770 (CYP83A1)	*A. thaliana*	Forward primer<br>AGGAACAACGGTCAACGTCA<br>Reverse primer<br>CGGTCCCCATTCTTTCTCGT

Table S2: Aligned reads for transcriptome profiling

Sample name	Aligned reads	Assigned Reads	Mapped Ratio
Ara_M2	34,670,703	31,998,776	92,3%
Ara_M3	31,050,325	28,078,404	90,4%
Ara_M4	29,883,629	26,930,049	90,1%
Ara_C1	33,833,716	27,677,748	81,8%
Ara_C2	30,911,854	24,741,132	80,0%
Ara_C3	30,653,084	24,528,34	80,0%
Ara_T1	29,290,278	25,109,740	85,7%
Ara_T2	32,381,425	28,898,406	89,2%
Ara_T3	32,197,738	28,344,993	88,0%

Sample name	Aligned reads	Assigned Reads	Mapped Ratio
Cam_M1	33,109,309	22,574,696	68,2%
Cam_M2	32,233,737	22,145,697	68,7%
Cam_M3	33,890,114	22,984,537	67,8%
Cam_C1	38,817,320	24,936,094	64,2%
Cam_C2	31,267,901	20,165,623	64,5%
Cam_C3	28,166,913	17,126,400	60,8%
Cam_T1	29,673,896	20,168,532	68,0%
Cam_T2	29,764,440	20,762,349	69,8%
Cam_T3	34,740,771	24,509,728	70,6%

Table S3: List of plant genes upregulated by aphid infestation alone (extracted from the RNA-seq data by Annacondia et al. [2021; doi: 10.1111/nph.17226]) but downregulated in plants concomitantly aphid-infested and infected by TuYV or CaMV.

		Annacondia et al.		This study			
		Aphid vs. Control		TuYV vs. Mock		CaMV vs. Mock	
**Gene locus**	**Gene**	**log2FC**	**P-adj**	**log2FC**	**P-adj**	**log2FC**	**P-adj**
AT1G54050	17.4 kDa class III heat shock protein [Source:UniProtKB/Swiss-Prot;Acc:Q9SYG1]	3.73	5.84E-11	-	-	-1.59	1.62E-12
AT4G17490	Ethylene-responsive transcription factor 6 [Source:UniProtKB/Swiss-Prot;Acc:Q8VZ91]	3.04	3.02E-08	-	-	-1.27	1.06E-16
AT4G23180	Cysteine-rich receptor-like protein kinase 10 [Source:UniProtKB/Swiss-Prot;Acc:Q8GYA4]	1.35	4.72E-02	-	-	-1.12	4.00E-13
AT5G47230	ERF5 [Source:UniProtKB/TrEMBL;Acc:A0A178UKK9]	2.97	2.94E-07	-	-	-1.12	4.30E-09
AT2G24600	Ankyrin repeat family protein [Source:UniProtKB/TrEMBL;Acc:F4IPR3]	2.62	1.69E-07	-	-	-1.04	8.01E-06
AT3G18710	RING-type E3 ubiquitin transferase [Source:UniProtKB/TrEMBL;Acc:A0A178VJJ8]	1.83	1.83E-02	-	-	-1.00	2.84E-12
AT1G33600	Leucine-rich repeat (LRR) family protein [Source:UniProtKB/TrEMBL;Acc:Q9FW48]	1.42	4.79E-02	-	-	-0.97	6.95E-13
AT1G50750	Plant mobile domain protein family [Source:TAIR;Acc:AT1G50750]	3.84	6.82E-11	-	-	-0.95	5.42E-06
AT2G29500	17.6 kDa class I heat shock protein 2 [Source:UniProtKB/Swiss-Prot;Acc:Q9ZW31]	2.89	7.66E-06	-	-	-0.95	4.48E-02
AT1G19770	PUP14 [Source:UniProtKB/TrEMBL;Acc:A0A178WHV5]	1.55	5.69E-03	-	-	-0.88	2.14E-10
AT3G55840	Nematode resistance protein-like HSPRO1 [Source:UniProtKB/Swiss-Prot;Acc:Q9LY61]	2.09	1.79E-02	-	-	-0.84	2.57E-09
AT2G26530	AR781 [Source:UniProtKB/TrEMBL;Acc:A0A178VPT1]	2.04	4.30E-04	-	-	-0.84	5.41E-09
AT1G56240	F-box protein PP2-B13 [Source:UniProtKB/Swiss-Prot;Acc:Q9C7J9]	1.96	3.46E-02	-	-	-0.79	5.01E-08
AT1G73080	Leucine-rich repeat receptor-like protein kinase PEPR1 [Source:UniProtKB/Swiss-Prot;Acc:Q9SSL9]	1.77	5.00E-02	-	-	-0.76	2.03E-03
AT5G45340	Abscisic acid 8'-hydroxylase 3 [Source:UniProtKB/Swiss-Prot;Acc:Q9FH76]	3.69	5.91E-12	-	-	-0.72	1.54E-08
AT2G06050	12-oxophytodienoate reductase 3 [Source:UniProtKB/Swiss-Prot;Acc:Q9FUP0]	1.48	3.40E-02	-	-	-0.68	7.44E-05
AT5G46510	Disease resistance protein (TIR-NBS-LRR class) family [Source:UniProtKB/TrEMBL;Acc:Q9FHF4]	2.24	3.57E-03	-	-	-0.67	2.53E-02
AT1G09950	RAS1 [source:Araport11]	2.24	5.69E-03	-	-	-0.63	8.50E-04
AT1G30135	Protein TIFY 5A [Source:UniProtKB/Swiss-Prot;Acc:Q8LBM2]	1.96	3.68E-02	-	-	-0.63	1.12E-02
AT4G09150	T-complex protein 11 [Source:UniProtKB/TrEMBL;Acc:F4JJB2]	1.93	2.19E-03	-	-	-0.62	6.87E-04
AT3G56880	VQ motif-containing protein [Source:UniProtKB/TrEMBL;Acc:Q9LES0]	2.21	3.08E-05	-	-	-0.62	1.75E-08
AT5G58430	Exocyst complex component EXO70B1 [Source:UniProtKB/Swiss-Prot;Acc:Q9FGH9]	1.80	8.87E-03	-	-	-0.60	6.34E-06
AT1G74930	Ethylene-responsive transcription factor ERF018 [Source:UniProtKB/Swiss-Prot;Acc:Q9S7L5]	4.17	1.10E-15	-	-	-0.60	1.56E-03
AT4G24380	At4g24380 [Source:UniProtKB/TrEMBL;Acc:Q8GX45]	2.76	1.14E-06	-	-	-0.59	3.24E-05
AT3G57530	Calcium-dependent protein kinase 32 [Source:UniProtKB/Swiss-Prot;Acc:Q6NLQ6]	2.81	5.94E-08	-	-	-0.58	1.15E-04
AT1G57990	PUP18 [Source:UniProtKB/TrEMBL;Acc:A0A178WC51]	1.85	4.39E-03	-	-	-0.55	2.81E-05

AT1G33760	Ethylene-responsive transcription factor ERF022 [Source:UniProtKB/Swiss-Prot;Acc:Q9LQ28]	4.18	1.62E-12	-	-	-0.55	1.91E-03
AT1G72910	Similar to part of disease resistance protein [Source:UniProtKB/TrEMBL;Acc:Q9SSN5]	1.83	8.89E-04	-	-	-0.55	4.14E-03
AT1G72920	Similar to part of disease resistance protein [Source:UniProtKB/TrEMBL;Acc:Q9SSN4]	3.11	1.03E-08	-	-	-0.54	1.69E-02
AT5G61600	Ethylene-responsive transcription factor ERF104 [Source:UniProtKB/Swiss-Prot;Acc:Q9FKG1]	3.44	1.55E-11	-	-	-0.48	2.50E-04
AT1G19380	At1g19380 [Source:UniProtKB/TrEMBL;Acc:Q9LN60]	2.13	1.35E-03	-	-	-0.47	1.21E-03
AT1G02660	Phospholipase A1 PLIP2, chloroplastic [Source:UniProtKB/Swiss-Prot;Acc:F4HXL0]	1.76	2.46E-02	-	-	-0.44	4.18E-02
AT2G38470	WRKY33 [Source:UniProtKB/TrEMBL;Acc:A0A384L4W4]	2.45	1.73E-07	-	-	-0.40	1.05E-02
AT1G07135	At1g07135 [Source:UniProtKB/TrEMBL;Acc:Q9LMK6]	2.95	2.90E-08	-	-	-0.39	5.02E-03
AT4G17230	SCARECROW-like 13 [Source:TAIR;Acc:AT4G17230]	1.38	4.52E-02	-	-	-0.39	2.46E-02
AT4G11280	1-aminocyclopropane-1-carboxylate synthase 6 [Source:UniProtKB/Swiss-Prot;Acc:Q9SAR0]	3.56	3.05E-14	-	-	-0.32	2.13E-02
AT1G21910	DREB26 [Source:UniProtKB/TrEMBL;Acc:A0A178W3Q9]	2.16	7.49E-04	-0.62	1.00E-02	-	-

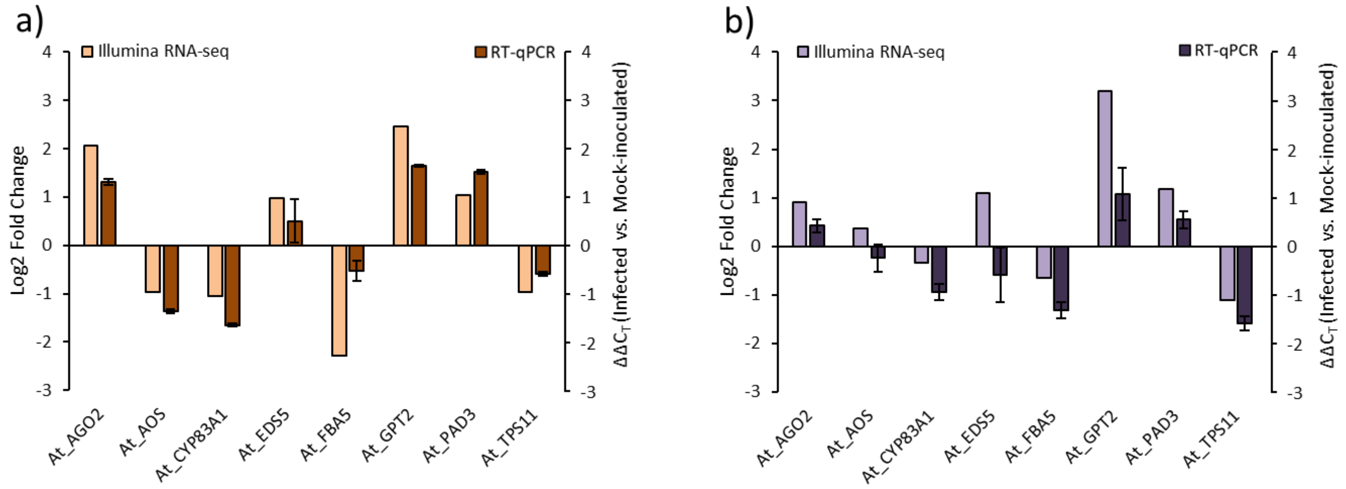

Figure S1. Validation of Illumina RNA-seq expression data by quantitative reverse-transcription PCR (RT-qPCR). a) CaMV-infected Arabidopsis. b) TuYV-infected Arabidopsis. The y-axis presents the normalized log2 fold change of expression derived from Illumina RNA-seq read counts and PCR $\Delta\Delta C_T$, respectively. The TAIR gene loci of the tested mRNAs are: At_AGO2, AT1G31280; At_AOS, AT5G42650; At_CYP83A1, AT4G13770; At_EDS5, AT4G39030; At_FBA5, AT4G26530; At_GPT2, AT1G61800; At_PAD3, AT3G26830; At_TPS11, AT2G18700.

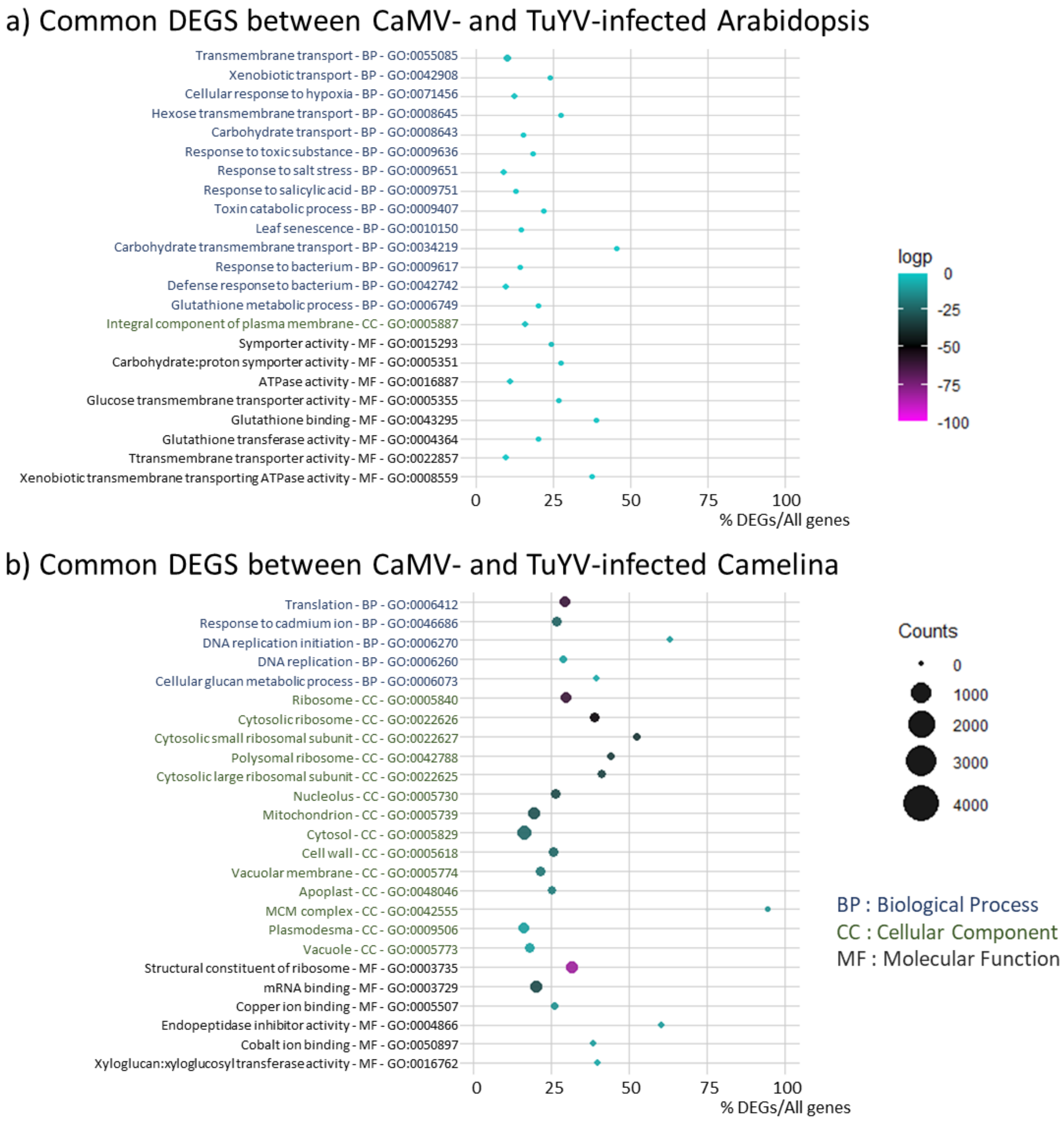

Figure S2: Gene ontology analysis showing the Top 25 GO of deregulated processes. a) common DEGS (n=956) between CaMV-infected and TuYV-infected Arabidopsis and b) common DEGS (n=6,692) between CaMV-infected and TuYV-infected Camelina. GO IDs and corresponding GO terms are specified in the vertical axis. For each category (BP: Biological Process, CC: Cellular Component and MF: Molecular Function), GOs are sorted according to decreasing log2 (1/p-value), also indicated by the color of each spot, in order to place most significantly enriched GOs on top of the graph. The absolute number of DEGs that matched the GO term is indicated by the size of each spot, whereas the horizontal axis shows the ratio of DEGs vs. all genes belonging to the GO term.

Supplementary heatmaps

Callose deposition is induced via pathogen molecular pattern- and pathogen effector-triggered immunity pathways. We found that expression of genes related to callose deposition was only slightly deregulated in aphid-infested infected plants except for a strong upregulation of *SWEET13* in Camelina infected with CaMV or TuYV (log2FC > 3.4), and a slight downregulation of *BHLH89* in CaMV-infected Arabidopsis and Camelina. SWEET13 is a sugar transporter and there is no direct link with callose (at least I did not find any, even if there is callose deposition in the Arabidopsis GO annotation). BHLH89 is a transcription factor that is upstream of callose deposition; (no more information). *UGP1* was virus-specifically upregulated in TuYV-infected Camelina (log2FC >1.10) and Arabidopsis (log2FC = 0.63) and downregulated in CaMV-infected plants (log2FC < -0.5). *UGP1* (AT3G03250) is a UDP-glucose pyrophosphorylase and involved in the first synthesis steps of cellulose and other sugar polymers (PMID 29569779) such as callose.

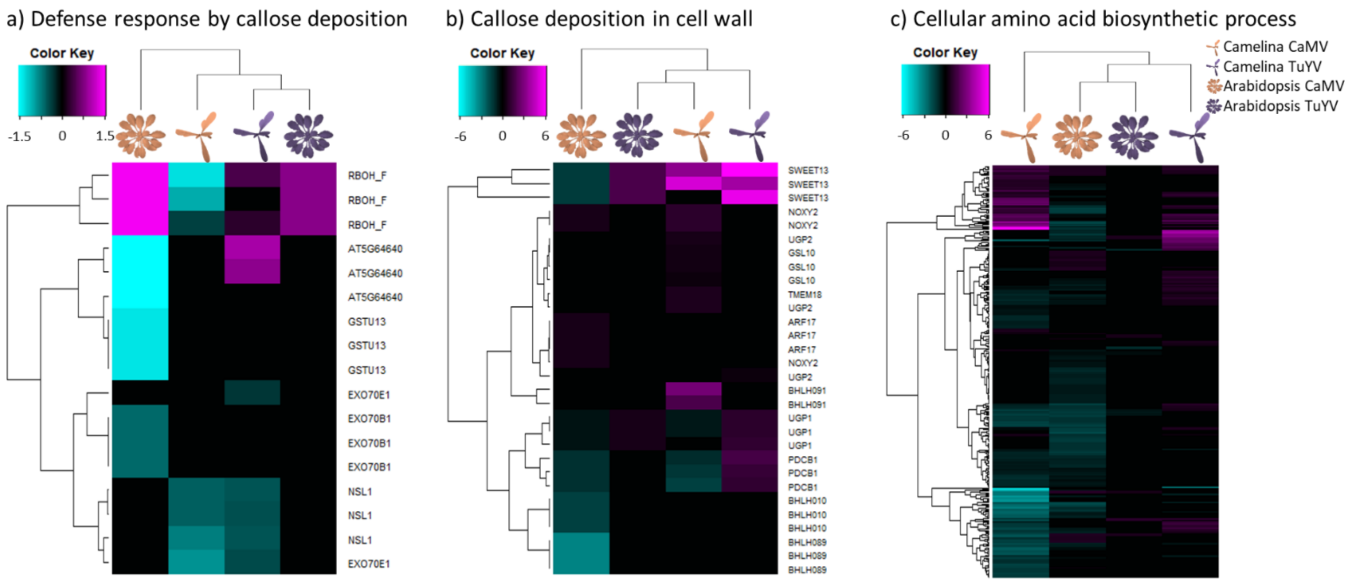

Figure S3. Hierarchical clustering of differentially expressed genes (DEGs) related to a) Defense response by callose deposition (GO:0052542), b) Callose deposition in cell wall (GO:0052543) and c) Cellular amino acid biosynthetic process (GO:0008652) in CaMV- and TuYV-infected *Arabidopsis thaliana* and *Camelina sativa* compared to their mock-inoculated relatives (Supplementary Dataset S2). The color keys show log2fold changes as indicated below the keys in gradients from the minimal value in cyan to the maximal value in magenta.

Supplementary sequence information S1. Complete genome sequences of CaMV CM1841Rev and TuYV FL1 isolates reconstructed by RNA-seq. The nucleotides highlighted in yellow represent single nucleotide polymorphism (SNP) positions distinguishing the reconstructed sequences from the reference sequences of CaMV strain CM1841 (V00140) and TuYV isolate BWYV-FL1 (X13063).

```
>CM1841rev_RNAseq_consensus
GGTATCAGAGCCATGAATCGGTTTAAAAACCAAACTCAAGAGGGTAAAACCTCACCAAAATACGAAAGAGTTCTTAACTCTAAAGATAAAAGATCTTTCAAGATCAA
AACTAGTTCCCTCACACCGGTGACCGACAGGTTTACCACCGTAAGGTTTCAGAACAACATCGAATGCGTTTACGCCAACTTCGACTCTCAGCTCAAGTCGTCGTACG
ATGGTAGATCTAAAAAGATCAAGAATCTAAGCCTTAAAAATCTTAGATGTTATGAAGCCTTCCTCAGGAAGTACCTTCTGGAACAATAAATCTCTCTGAGAATAGTA
CTCTAACGAGTATCCACAGGAAAAATAATCTTCTGTGTTGAGATGGATTTGTATCCAGAAGAAAATACCCAAAGCGAGCAATCGCAGAATTCTGAAAATAATATGCA
AATATTTAAGTCAGAAAATTCGGATGGATTCTCCTCCGATCTAATGATCTCAAACGATCAATTAAAAAATATCTCTAAGACCCAATTAACTTTGGAGAAAGAAAAGA
TATTTAAAATGCCTAACGTTTTATCTCAAGTTATGAAAAAAGCGTTTAGCAGGAAAAACGAGATTCTTTACTGCGTCTCGACAAAAGAATTATCAGTGGACATTCAC
GATGCCACAGGTAAGGTATATCTCCCTTTAATCACTAGAGAGGAGATAAATAAAAGACTTTCTAGCTTAAAACCTGAAGTCAGAAAGATCATGTCCATGGTTCATCT
TGGAGCGGTCAAAATATTGCTTAAAGCTCAATTTCGAAATGGGATTGATACCCCAATCAAAATTGCTTTAATCGATGATAGAATTAATTCTAGAAGAGATTGCCTTC
TCGGTGCAGCCAAAGGTAATCTAGCATACGGTAAGTTTATGTTTACTGTATACCCCAAGTTTGGAATAAGCCTTAATACCCAAAGACTTAACCAAACCTTAAGCCTT
ATTCATGATTTTGAGAATAAAAATCTTATGAATAAAGGTGATAAAGTTATGACCATAACCTATATCGTAGGATATGCATTAACTAATAGTCATCATAGCATAGATTA
TCAATCGAATGCTACAATTGAACTAGAAGACGTATTTCAAGAAATTGGAAATGTCCAGCAATCTGACTTTTGTACAATACAAAATGACGAATGCAATTGGGCCATTG
ATATAGCCCAAAACAAAGCCTTATTAGGAGCTAAAACCCAATCCCAAATTGGTAATAGTCTTCAAATAGGAAACAGTGCTTCATCCTCTAATACTGAAAATGAATTA
GCTAGGGTAAGCCAAAACATAGATCTTTTAAAAAACAAATTAAAAGAGATCTGTGGAGAATAAAATGAGCATTACGGGTCAACCGCATGTTTATAAAAAGGATACTA
TTATTAGACTAAAACCATTGTCTCTTAATAGTAATAATAGAAGTTATGTTTTTAGTTCCTCAAAAGGGAACATTCAAAATATAATTAATCATCTTAACAACCTCAAT
GAGATTGTAGGAAGAAGCTTACTCGGAATATGGAAGATCAACTCATACTTCGGACTAAGCAAAGACCCTTCGGAGTCCAAATCAAAAAACCCGTCAGTTTTTAATAC
TGCAAAAAACATTTTTAAAAGTGGGGGGGTTGATTACTCGAGCCAACTAAAGGAAGTAAAATCCCTTTTAGAAGCTCAAAATACTAGAATTAAAAATCTAGAAAATG
CAATTCAATCCTTAGATAATAAGATTGAACCAGAGCCCTTAACTAAAGAAGAAGTTAAAGAGCTAAAAGAATCGATTAACTCGATCAAAGAAGGATTAAAGAATATT
ATTGGCTGAAATGGCTAATCTTAATCAAATCCAGAAAGAAGTCTCTGAAATCCTCAGTGACCAAAAATCCATGAAATCGGATATAAAAGCTATCTTAGAATTGCTAG
GATCCCAAAATCCTACTAAAGAAAGCTTAGAAGCCGTTGCAGCGAAAATCGTTAATGACTTAACCAAGCTCATCAATGATTGTCCTTGTAACAAAGAGATATTAGAA
GCCTTAGGCAATCAGCCTAAAGAGCAACTAATAGAACAACCTAAAGAAAAAGGCAAAGGCCTTAATCTAGGAAAATATACTTACCCCAATTACGGCGTAGGAAATGA
AGAATTAGGATCCTCTGGAAACCCTAAAGCTTTAACTTGGCCTTTCAAAGCTCCAGCAGGATGGCCGAATCAATTTTAGACAGGACCATTAACCGGTTCTGGTATAA
CCTGGGAGAAGATTGTCTCTCAGAAAGTCAATTTGACCTTATGATAAGGTTAATGGAAGAGTCCCTTGACGGGGACCAAATTATTGATCTAACCTCTCTACCTAGTG
ATAATTTGCAGGTCGAACAGGTTATGACAACTACCGACGACTCGATCTCGGAAGAATCAGAATTCCTTCTAGCAATAGGAGAAATATCTGAAGACGAAAGTGATTCA
GGAGAAGAACCTGAATTCGAACAAGTTCGAATGGATCGAACAGGAGGAACGGAGATTCCCAAAGAAGAAGATGGTGAAGGACCATCTAGATACAATGAGAGAAAGAG
AAAGACCCCGGAGGACCGGTACTTTCCAACTCAACCAAAGACCATCCCAGGACAAAAGCAAACGTCTATGGGAATGCTCAACATTGACTGCCAAATCAATCGAAGAA
CTTTAATCGATGATTGGGCAGCAGAAATCGGATTGATAGTCAAAACCAACAGAGAAGACTATCTTGATCCAGAAACAATACTACTCTTGATGGAACACAAAACATCA
GGAATAGCCAAGGAGTTAATCCGAAATACAAGATGGAACCGTACTACCGGCGATATCATAGAACAGGTGATCAATGCAATGTACACCATGTTCTTAGGACTTAACTA
CTCCGACAACAAGGTTGCTGAAAAGATAGACGAGCAAGAGAAGGCCAAGATCAGAATGACCAAGCTCCAGCTCTTCGACATCTGCTACCTTGAAGAATTTACATGTG
ATTATGAGAAGAACATGTACAAGACGGAAATGGCGGATTTCCCTGGATACATCAACCAGTACCTGTCAAAAATCCCCATCATAGGAGAAAAAGCGCTAACACGCTTT
AGGCATGAAGCCAACGGAACCAGCATCTACAGCTTAGGTTTCGCGGCAAAGATAGTAAAAGAAGAACTATCAAAAATCTGCGACTTATCAAAGAAGCAGAAGAAGTT
GAAGAAATTCAACAAGAAATGCTGCAGCATCGGTGAAGCTTCAGTAGAATATGGAGGCAAGAAAACATCCAAGAAGAAGTATCATAAGCGATACAAGAAAAGATATA
AGGTCTATAAACCTTATAAGAAGAAGAAGAAATTCCGATCCGGAAAATACTTCAAGCCCAAAGAGAAGAAGGGCTCAAAGCGAAAGTATTGCCCAAAAGGCAAGAAG
GACTGCAGATGTTGGATCTGCAATATCGAAGGCCATTACGCCAACGAATGTCCTAATCGACAAAGCTCGGAGAAGGCTCACATCCTTCAACAAGCAGAGAATTTGGG
TCTCCAGCCCGTTGAAGAACCCTATGAAGGAGTTCAAGAAGTATTCATCTTAGAATACAAAGAAGAGGAAGAAGAAACCTCTACAGAAGAAAGCGATGATGAATCAT
CTACTTCTGAAGACTCAGACTCAGATTGAGCAGGTGATGAACGTCACCAATCCCAATTCGATCTACATCAAGGGAAGACTCTACTTCAAAGGATACAAGAAGATAGA
GCTTCACTGTTTTGTAGACACGGGAGCAAGCTTATGCATAGCATCCAAGTTCGTCATACCAGAAGAACATTGGGTTAATGCAGAAAGACCAATAATGGTCAAAATAG
CAGATGGAAGTTCAATTACCATCAGCAAAGTCTGCAAAGACATAGACTTGATCATAGCCGGCGAGATATTCAAAATTCCCACCGTCTATCAGCAAGAAAGTGGCATC
GATTTCATAATCGGCAACAACTTTTGTCAACTGTATGAACCATTCATACAGTTTACAGATAGAGTTATCTTCACAAAGAACAAGTCCTATCCTGTTCATATTACGAA
GCTAACAAGAGCAGTGCGAGTAGGCATCGAAGGATTTCTTGAATCAATGAAGAAACGTTCAAAGACTCAGCAACCTGAGCCGGTGAACATTTCGACAAACAAGATAG
AAAATCCACTAGAAGAAATTGCTATTCTTTCAGAGGGGAGGAGGTTATCAGAAGAAAAACTTTTCATCACTCAACAAAGAATGCAAAAAATCGAAGAACTACTAGAG
AAAGTATGTTCAGAAAATCCATTAGATCCTAACAAGACTAAGCAATGGATGAAAGCTTCAATCAAGCTCAGCGACCCAAGCAAAGCTATCAAGGTTAAACCCATGAA
ATACAGCCCAATGGATCGTGAAGAATTTGACAAGCAAATCAAAGAGTTACTGGACCTTAAAGTCATTAAACCCAGTAAAAGCCCTCACATGGCACCAGCCTTCTTGG
TCAACAATGAAGCCGAGAAGCGAAGAGGAAAGAAACGTATGGTAGTCAACTACAAAGCTATGAACAAAGCCACCATAGGAGACGCATACAATCTTCCCAACAAAGAC
GAGTTACTTACACTCATTCGAGGAAAGAAGATCTTTTCTTCCTTCGACTGTAAGTCCGGATTCTGGCAAGTTCTACTTGATCAAGAATCAAGACCTCTAACGGCATT
CACATGTCCACAAGGTCACTACGAATGGAATGTGGTCCCTTTCGGCCTAAAGCAGGCACCATCCATATTCCAGAGACACATGGACGAAGCATTTCGTGTGTTCAGAA
AATTCTGTTGCGTGTATGTCGACGACATCCTCGTATTCAGTAACAACGAAGAAGATCACCTACTTCACGTAGCAATGATCTTACAAAAGTGCAATCAACATGGAATC
ATTCTTTCCAAGAAGAAAGCACAACTCTTCAAGAAGAAGATAAACTTCCTTGGTCTAGAAATAGATGAAGGAACACACAAGCCTCAAGGACATATCTTGGAACATAT
CAACAAATTCCCAGATACCCTTGAAGACAAGAAGCAACTTCAGAGATTCTTAGGCATCCTAACATATGCCTCTGATTATATCCCGAAGCTAGCTCAAATCAGAAAGC
CTCTGCAAGCCAAGCTTAAAGAAAATGTTCCATGGAAATGGACAAAGGAGGACACCCTCTACATGCAAAAGGTGAAGAAAAATCTGCAAGGATTTCCTCCACTACAT
CATCCCTTACCAGAGGAAAAGCTGATCATCGAGACCGACGCATCAGACGACTACTGGGGAGGTATGTTAAAAGCTATCAAAATTAACGAAGGTACTAATACCGAGTT
AATTTGCAGATACGCATCTGGAAGCTTTAAAGCTGCAGAAAGGAATTACCACAGCAATGACAAAGAGACATTGGCGGTAATAAATACTATAAAGAAATTCAGTATTT
ATCTAACTCCTGTTCATTTTCTGATTAGGACAGATAATACTCATTTCAAGAGTTTTGTTAACCTTAATTACAAAGGAGATTCAAAACTTGGAAGAAACATCAGATGG
CAAGCATGGCTTAGCCACTATTCGTTTGATGTTGAACATATTAAAGGAACCGACAACCACTTTGCGGACTTCCTTTCAAGAGAATTCAATAAGGTTAATTCCTAATT
GAAATCCGAAGATAAGATTCCCACACACTTGTGGCTGATATCAAAAAGGCTACTACCTATATAAACACATCTCTGGAGACTGAGAAAATCAGACCTCCAAGCATGGA
GAACATAGAAAAACTCCTCATGCAAGAGAAAATACTAATGCTAGAGCTCGATCTAGTAAGAGCAAAAATAAGCTTAGCAAGAGCTAACGGCTCTTCGCAACAAGGAG
ACCTCCCTCTCCACCGTGAAACACCGGTAAAAGAAGAAGCAGTTCATTCTGCACTGGCCACTTTTACGCCAACTCAAGTAAAGGCTATTCCAGAGCAAACGGCTCCT
GGTAAAGAATCAACAAATCCGTTGATGGCTAGTATCTTGCCAAAAGATATGAACCCAGTTCAAACTGGGATAAGGCTTGCAGTGCCAGGGGACTTTTTACGTCCTCA
TCAGGGAATTCCAATCCCACAAAAATCTGAGCTTAGCAGCATAGTTGCTCCTCTCAGAGCAGAATCGGGTATTCAACACCCTCATATCAACTACTACGTTGTGTATA
ACGGTCCACACGCCGGTATATACGATGACTGGGGTTGTACAAAGGCGGCAACAAACGGCGTTCCCGGAGTTGCACACAAGAAGTTTGCCACTATTACAGAGGCAAGA
GCAGCAGCTGACGCGTACACAACAAGTCAGCAAACAGACAGGTTGAACTTCATCCCCAAAGGAGAAGCTCAACTCAAGCCCAAGAGCTTTGCGAAGGCCTTAACCAG
CCCACCAAAGCAAAAAGCCCACTGGCTCACGCTAGGAACCAAAAGGCCCAGCAGTGATCCAGCCCCAAAAGAGATCTCCTTTGCCCCGGAGATCACCATGGACGACT
TTCTCTATCTCTACGATCTAGGAAGAAAGTTCGACGGAGAAGGTGACGATACCATGTTCACCACCGATAATGAGAAGATTAGCCTCTTCAATTTCAGAAAGAATGCT
GACCCACAGATGGTTAGAGAGGCCTACGCGGCAGGTCTCATCAAGACGATCTACCCGAGTAATAATCTCCAGGAGATCAAATACCTTCCCAAGAAGGTTAAAGATGC
AGTCAAAAGATTCAGGACTAACTGCATCAAGAACACAGAGAAAGATATATTTCTCAAGATCAGAAGTACTATTCCAGTATGGACGATTCAAGGCTTGCTTCATAAAC
CAAGGCAAGTAATAGAGATTGGAGTCTCTAAGAAAGTAGTTCCTACTGAATCAAAGGCCATGGAGTCAAAAATTCAGATCGAGGATCTAACAGAACTCGCCGTGAAG
ACTGGCGAACAGTTCATACAGAGTCTTTTACGACTCAATGACAAGAAGAAAATCTTCGTCAACATGGTGGAGCACGACACTCTCGTCTACTCCAAGAATATCAAAGA
TACAGTCTCAGAAGACCAAAGGGCTATTGAGACTTTTCAACAAAGGGTAATATCGGGAAACCTCCTCGGATTCCATTGCCCAGCTATCTGTCACTTCATCAAAAGGA
CAGTAGAAAAGGAAGGTGGCACCTACAAATGCCATCATTGCGATAAAGGAAAGGCTATCGTTCAAGATGCCTCTGCCGACAGTGGTCCCAAAGATGGACCCCCACCC
ACGAGGAGCATCGTGGAAAAAGAAGACGTTCCAACCACGTCTTCAAAGCAAGTGGATTGATGTGATATCTCCACTGACGTAAGGGATGACGCACAATCCCACTATCC
TTCGCAAGACCCTTCCTCTATATAAGGAAGTTCATTTCATTTGGAGAGGACACGCTGAAATCACCAGTCTCTCTCTACAAATCTATCTCTCTCTATTTTCTCCATAA
TAATGTGTGAGTAGTTCCCAGATAAGGGAATTAGGGTTCTTATAGGGTTTCGCTCACGTGTTGAGCATATAAGAAACCCTTAGTATGTATTTGTATTTGTAAAATAC
TTCTATCAATAAAATTTCTAATTCCTAAAACCAAAATCCAGTACTAAAATCCAGATCACCTAAAGTCCCTATAGATCTTTGTCGTGAATATAAACCAGACATGAGAC
GACTAAACCTGGAGCCCAGACGCCGTTCGAAGCTAGAAGTACCGCTTAGGCAGGAGGCCGTTAGGGAAAAGATGCTAAGGCAGGGTTGGTTACGTTGACTCCCCCGT
AGGTTTGGTTTAAATATGATAAAGTGGACGGAAGGAAGGAGGAAGACAAGGAAGGATAAGGTTGCAGGCCCTGTGCAAGGTAAGAAGATGGAAATTTGATAGAGGTA
CGTTACTATACCTATACTATACGCTAAGGGATGCTTGTATTTTACCCTATACCCCCTAATAACCCCTTATCGATTTTAAGAAATAATCCGCATAAGCCCCCGCTTAA
AAAATT
```

>TuYV_RNAseq_consensus
ACAAAAGAAACCAGGAGGGAATCCTTAGTTGATGCAATTTCTCGCTCACGATAACTTTCACACTTTGCAAGTCAAGAAAGTCCGATTCCTCCACCCTCAACAAGAAG
TGTTTCTTTTAGCAGGTTTATTGCTCAATATAAAACAATTcGTACGAGCAATCAAAGAGCGCAATAATGTATTCAAAATTGATGTTTTTCTTCGCTCTTTGCTCTAT
CAGCTTCCTTTTCACCTCGGAAGCTGCTTCCACGATGCTCCTCGAGAGCTCATACCTGCCACTGAACCAGAGTTATGCGCCTGGTTTTCTTTACAAACGGGATATGC
TCCCGCCTCCACTTCAGGCCGTGTTAACTTACACGTGCCCGGAACCAAGACCTCTCGCAGAAGAATCATACAACGATCTTTTGCGAGCGATTTCTCAGAAAAGCTCA
AGCGATTTCCAGAATGCTTATTCGTTAGCCTTGAGCTTTTCCAGCGACTTCTATCAACATGGACTAAAGACGTTGAAAGACGTATCTTTTTTAGCTGTCGAGAAATT
CCTCTGGGGTCTGACACGCTTATGGAGCTCGCTAATCTTGGCGAGTTTCTCCGCGTTATGGTGGTTGGTGAGCAATTTCACAACTCCCGTCTTCTGTCTCGCCTTGC
TGTACACTGTTACAAAATATATGGTGAAGACGGTTTCATTTCTTTTTGGAGGATTGCCAATCTGGATCATTTCGATTGCTTTCTCACTCCTGAAGAAATCCTTTTCA
GCTCTTCGGTCTACACCGAAATGTTTGTATGAAAAGGCCATAGACGGTTTCAAGAGTTTCACTATCCCGCAGAGTCCCCCAAAATCTTGCGTGATTCCCATCACCCA
CGCAAGCGGAAACCACGCTGGCTATGCCAGTTGTATCAAGCTATATAACGGAGAAAATGCTCTAATGACGGCAACTCACGTCCTACGTGATTGCCCCAACGCCGTGG
CTGTTTCCGCCAAAGGACTCAAAACGCGGATTCCACTCGCAGAATTCAAAACAATCGCGAAATCCGACAAAGGTGATGTTACCCTCCTTCGCGGCCCCCCCAATTGG
GAAGGACTGTTGGGCTGTAAAGCGGCCAACGTTATAACAGCTGCcAACCTAGCGAAATGCAAAGCATCCATATACTCTTTTGACAGAGATGGCTGGGTTAGCAGTTA
TGCCGAGATCGTgGGCTCAGAAGGTACAGATGTTATGGTTCTGAGCCATACGGAAGGAGGACACTCCGGAAGCCCCTACTTCAATGGTAAAACCATCTTGGGGGTTC
ATTCAGGTGCCAGTGCTACTGGAAATTACAATTTAATGGCACCAATCCCATCCCTCCCCGGACTTACTAGTCCGACTTATGTGTTCGAAACCACCGCACCACAAGGA
AGAGTTTTCGCACAAGAAGATATCGCTGAAATCGAAGGCCTCTATGCACAAGTAATGAAAAGAGTTCAACAAGCGGAAGATTTCAAACCCAAAACTGGAAAGTATTG
GGGTGATATGGAGGATGATGAAGACATTTTCTTCGAAAGCAAAGAAGATCTGTCGGGAAACGGAGTGCGCGGCACCGTCCGCGGAACAAACGGAGAAGGCAGCTCCA
CCCCAAAGACAAGCAACGTCGATGGGAAAGAGATGATGGAGAAAATAATCTCATCTCTAGTGGGAAAGATAAATCTCGAGAACATCGAGAGGAAAGTGATAGAGGAG
ATCTCCGCGAAAGCGATGAAAACTCCGAAATCCCGCCGCAGAAGAGCCCCAAAGAAACAGCCGGAGAGTTCGAAAGATACTTCTCCTCGCTCTACAACTGGGAAGTA
CCAACCTCCCCACGTGAGGTCCCCGGCTTCCGTCACTGCGGCAAACTGCCCCAATACTACCACCCCAAGCAAAAAGAAGAATCTAGCTGGGGGAAGACCCTCGTCGG
GAACCATCCCGCGTTGGGTGAGAAAACAAGCGGCTTCGGCTGGCCCAAGTTCGGCCCCGAAGCAGAACTGAAGAGCCTGCGACTGCAGGCTTCACGGTGGCTGGAAC
GCGCCCAGTCCGCAGAAATACCCTCTGACGCTGAGAGGGAGCGTGTGATTCAAAAGACCGCAGATGTTTACCATCCTTGCCAAACAAATGGGCCTGCGGCAACCCGA
GGAGGAACACTAACCTGGAACAACTTCATGATTGATTTCAAACAGGCAGTGTTCTCGCTGGAGTTCGATGCCGGAATCGgcgTCCCCTATATTGCCTATGGCAAGCC
CACACACCGTGGGTGGGTTGAAGACCAGAAACTCCTTCCAATCCTAGCTCAATTGACCTTCTTCCGACTACAGAAGATGTTGGAGGTCAATTTCGAAGATATGGGAC
CTGAGGAGCTGGTCCGGAACGGTTTGTGTGATCCCATCCGATTATTCGTGAAGGGTGAGCCGCACAAGCAAGCGAAGCTCGATGAAGGCCGCTACCGCCTCATAATG
AGCGTTTCCCTCGTGGATCAACTGGTAGCCCGGGTTCTGTTTCAAAATCAGAACAAGCGGGAAATTGCCCTGTGGAGGGCCATCCCCAGCAAACCCGGTTTTGGCTT
GTCTACGGATGAGCAAGTGCTGGACTTTGTGGAAAGTCTGGCCCGTCAAGTAGGCACCACTACGACAGAGGTGGTTGCCAATTGGAAcAATTACTTGACGCCCACGG
ATTGCTCCGGTTTTGACTGGAGTGTTGCGGATTGGATGCTTCACGACGACATGATCGTCCGCAACAGACTTACCATCGACCTCAACCCCGCTACAGAAAGATTAAGA
TCTTGCTGGTTGAGGTGCATTTCAAACTCAGTATTGTGCCTGAGTGATGGCACCCTTTTAGCCCAAATTCATCCGGGCGTTCAGAAGAGTGGGAGCTATAATACATC
AAGCTCCAACTCCCGGATCCGAGTTATGGCCGCCTTCCACACAGGTGCCATCTGGGCTATGGCGATGGGTGATGATGCCCTCGAGTCCAATCCCGCTGACCTAGCAG
CGTACAAGAAACTAGGTTTCAAGGTTGAGGTTTCCGGACAACTGGAATTCTGCTCTCAcATCTTTAGAGCGCCGGACCTCGCCCTCCCTGTGAACGAAAATAAGATG
ATCTACAAACTGATCTATGGCTATAATCCAGGGAGCGGAAACGCTGAGGTAGTTTCAAACTACTTGGCCGCTTGTTTCTCAGTTCTGAACGAGTTGCGGCATGATCC
AGCGTCCGTTGAACTTCTTTACTCGTGGTTAGTCGATCCGGTGCTACCACAAAAGATACCAGGAGAGTAAAGAAGAAGAAAGTCAGCTTACATTGAAATTTTAAAGA
GGTTTCTGCAACAGTAAGAGACTTAAGCAAACCCAATTAAAGATACAACGGATTACAAATTCCTAGCAGGCTTCGCCGCAGGCTTCGTTTCATCGATACCAATATCC
GTGATCAGTATCTATTTCATCTACCTAAGAATCTCCAAACACGTACGCGAAATCGTTAATGAATACGGTCGTGGGTAGGAGAATTATCAATGGAAGAAGACGACCAC
GCAGGCAAACACGACGCGCTCAGCGCCCTCAGCCAGTGGTTGTGGTCCAAACCTCTCGGGCAACACAACGCCGACCTAGACGACGACGAAGAGGTAACAACCGGACA
GGAAGAACTGTTCCTACCAGAGGAGCAGGTTCGAGCGAGACATTTGTTTTCTCAAAAGACAATCTCGCGGGAAGTTCCAGCGGAGCAATCACGTTCGGGCCGAGTCT
ATCAGACTGCCCGGCATTCTCTAATGGAATGCTCAAGGCCTACCATGAGTATAAAATCTCAATGGTCATTTTGGAGTTCGTCTCCGAAGCCTCTTCCCAAAATTCCG
GTTCCATCGCTTACGAGCTGGACCCACACTGTAAACTCAACTCCCTTTCCTCAACTATCAACAAGTTCGGGATCACAAAGCCCGGGAAAAGGGCGTTTACAGCGTCT
TACATCAACGGAACGGAATGGCACGACGTTGCCGAGGACCAATTCAGGATCCTCTACAAAGGCAATGGTTCTTCATCGATAGCTGGTTCTTTCAGAATCACCATTAA
GTGTCAATTCCACAACCCCAAATAGGTAGACGAGGAACCCGGCCCTAGCCCAGGGCCTTCTCCCTCTCCACAACCCACACCCCAAAAGAAATATCGTTTTATCGTCT
ATACTGGAGTCCCCGTGACTCGTATAATGGCTCAATCTACGGATGATGCCATCTCTTTGTATGATATGCCGTCCCAACGGTTTCGCTACATAGAGGACGAGAACATG
AACTGGACGAACCTCGATTCTCGATGGTATTCCCAGAATTCTTTGAAAGCCATCCCGATGATAATAGTGCCAGTCCCTCAAGGTGAGTGGACCGTGGAAATATCGAT
GGAGGGGTATCAACCAACCTCAAGCACCACAGATCCTAACAAGGACAAACAAGATGGTCTCATCGCCTACAACGATGATCTTAGTGAAGGTTGGAACGTGGGGATTT
ACAATAATGTGGAGATAACCAACAACAAGGCCGATAATACTTTGAAGTATGGCCACCCAGACATGGAACTCAATGGCTGTCATTTCAATCAAGGACAGTGTCTGGAA
AGAGATGGAGATTTGACTTGTCATATCAAGACGACTGGTGACAATGCCTCCTTCTTTGTTGTTGGACCCGCTGTCCAGAAGCAATCTAAATATAATTACGCCGTTTC
GTACGGAGCCTGGACAGATCGGATGATGGAGATAGGGATGATCGCCATAGCACTTGATGAACAAGGCTCATCCGGTTCCGTAAAGACAGAAAGACCAAAGAGAGTTG
GGCACTCCATGGCAGTCTCAACCTGGGAGACTATAAAATTGCCGGAGAAGGGAAACTCCGAGGGATACGAAACCAGTCAAAGACAAGACTCTAAAACTCCTCCCACA
GCTAGTGGGGGTTCTGACACGCTGGACGTCGAAGAAGGAGGCTTGCCCCTTCCTGTTGAAGAAGAGATCCCCGATTTTGTTGGGGATAACCCCTGGTCTGACTTATC
GACTAAGAATTCACAGGAAGAAGAGGCTATGTCATCAGAGAGTGGTCTTAGACCCCAGTTGAAGCCTCCTGGTCTGCCAAAACCTCAACCGATCAGAACGATTCGAA
ACTTCGATCCAACACCGGATTTGGTTGAAGCGTGGCGACCCGATGTGAACCCCGGATATTCCAAAGCAGATGTGGCAGCCGCTACTATCATCGCCGGGGGTTCCATC
AAAGACGGCCGTTCTATGATTGATAAACGAAATAAAGCTGTGTTAGACGGTCGCAAGAGTTGGGGTTCCTCCTTGGCTTCCTCCCTCACGGGTGGTACGCTCAAGGC
CTCCGCCAAGTCGGAGAAGCTTGCCAAACTTACCACGAGTGAAAGGGCAAGGTATGAACGGATTAAGCGTCAGCAAGGCTCCACAAGAGCCTCGGAATTCCTAGAAT
CACTTCTAGCTGGCGAAGACCCCGACTCAAGGTTCTGAAGGGATACAACCTGACCCTTCCCGGTCCAGATGAACCCGTCCAAATCATCATCGTCAAGCCAGGGACTT
TAAACTGGAACGAATCCGTTTTACGGATAGGCAACGAGTGTTTTACGCTGGGAGAAATCCCTACGGCACTTCGGTGT
